# Structure of the Bacterial Sex F Pilus Reveals an Assembly of a Stoichiometric Protein-Phospholipid Complex

**DOI:** 10.1016/j.cell.2016.08.025

**Published:** 2016-09-08

**Authors:** Tiago R.D. Costa, Aravindan Ilangovan, Marta Ukleja, Adam Redzej, Joanne M. Santini, Terry K. Smith, Edward H. Egelman, Gabriel Waksman

**Affiliations:** 1 Institute of Structural and Molecular Biology, University College London and Birkbeck, Malet Street, London WC1E 7HX, UK; 2 BSRC, School of Biology, University of St Andrews, St Andrews KY16 9AJ, UK; 3 Department of Biochemistry and Molecular Genetics, University of Virginia, Charlottesville, VA 22908, USA

**Keywords:** Pilus, Conjugation, Type 4 Secretion System, Phospholipid, Protein-phospholipid complex, Structural Biology, Cryoelectron Microscopy

## Abstract

Conjugative pili are widespread bacterial appendages that play important roles in horizontal gene transfer, in spread of antibiotic resistance genes, and as sites of phage attachment. Among conjugative pili, the F “sex” pilus encoded by the F plasmid is the best functionally characterized, and it is also historically the most important, as the discovery of F-plasmid-mediated conjugation ushered in the era of molecular biology and genetics. Yet, its structure is unknown. Here, we present atomic models of two F family pili, the F and pED208 pili, generated from cryoelectron microscopy reconstructions at 5.0 and 3.6 Å resolution, respectively. These structures reveal that conjugative pili are assemblies of stoichiometric protein-phospholipid units. We further demonstrate that each pilus type binds preferentially to particular phospholipids. These structures provide the molecular basis for F pilus assembly and also shed light on the remarkable properties of conjugative pili in bacterial secretion and phage infection.

## Highlights

• The atomic structures of the F pilus and an F-like pilus are solved by cryo-EM
• The structure reveals an assembly of a protein-phospholipid complex unit
• Mutations at subunit-lipid interfaces affect phage infection and conjugation
• The structure elucidates the molecular basis for DNA transfer and phage attachment

## Graphical Abstract

**Figure:**
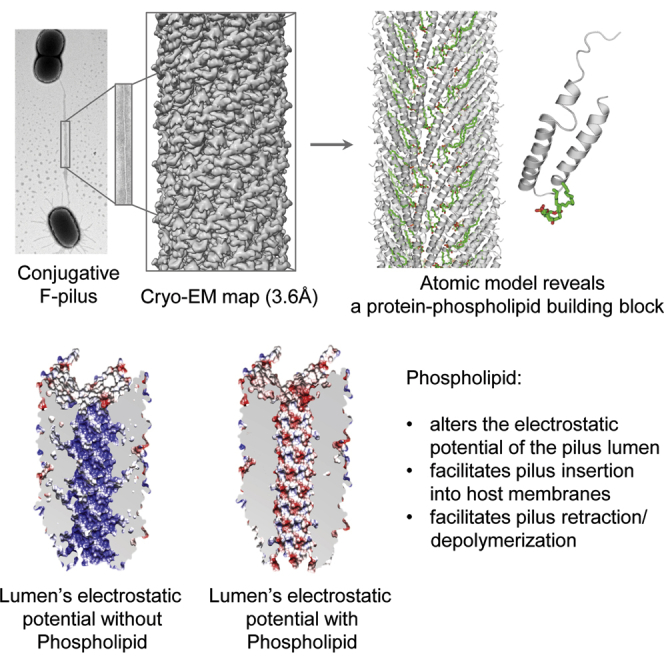

## Teaser

Conjugative pili, the appendages that enable DNA transfer between bacteria, are not made only of protein but, as near-atomic resolution cryoEM reveals, are assemblies of stoichiometric protein-phospholipid units, with the phospholipid component suited to electrochemically facilitating DNA transport.

## Introduction

Conjugation is the process by which genetic materials, notably plasmid DNAs, are transferred from one bacterium to another (Lederberg and Tatum, 1946). It is responsible for horizontal gene transfer among bacteria and is the primary means by which antibiotic resistance genes spread among bacterial populations (Thomas and Nielsen, 2005). Conjugation is mediated by a type IV secretion (T4S) system, a versatile secretion machine, operating in both Gram-negative and -positive bacteria and capable of secreting not only nucleic acids during conjugation, but also protein effectors and toxins during bacterial pathogenesis (Costa et al., 2015). Conjugative T4S systems in Gram-negative bacteria are composed of 12 components, termed VirB1-11 and VirD4, which form (1) a multi-megaDalton assembly embedded in the cell’s double lipid membrane, and (2) a pilus that extends to the cell surface. The membrane-embedded complex consists of an outer-membrane complex made of VirB7, VirB9, and VirB10 and a bi-partite inner-membrane complex made of VirB3, VirB4, VirB5, VirB6, VirB8, and VirB11 (Fronzes et al., 2009, Low et al., 2014, Rivera-Calzada et al., 2013). The pilus is a polymer of the VirB2 protein (or pilin). Three ATPases power the system: VirB4, VirB11, and VirD4, with VirD4 known as the “coupling protein” (CP) because it couples recruitment of the substrate to its delivery to the VirB transport machinery. The substrate itself is a protein-DNA complex in which the proteinaceous component is a protein, termed “relaxase,” that binds specifically to an “origin of transfer” (*oriT*) sequence on the plasmid DNA, nicks it, and covalently attaches to the 5′ end of the nicked strand (the T strand) (Ilangovan et al., 2015, Larkin et al., 2003). The covalent relaxase-DNA complex is then recruited to the T4S system by VirD4, transported through the machinery, and then through the pilus, which forms a tube that can deliver DNA to a recipient cell located at some distance away (Babic et al., 2008). The pilus is a dynamic structure that can depolymerize to bring donor and recipient cells closer to one another (Clarke et al., 2008, Novotny and Fives-Taylor, 1974).

The F plasmid has a remarkable status in the history of the fields of molecular biology and genetics. The F plasmid is not only able to conjugate itself from a donor cell to a recipient cell (it indeed encodes all the T4S system and relaxosome components) (Lawley et al., 2003), but also, by virtue of it being able to integrate within the genome of *Escherichia coli*, is able to conjugate the entire *E. coli* genome. This property was used to map the entire *E. coli* genome in the 1950s and 1960s, leading to seminal discoveries in genome organization, dynamics, and expression (Taylor and Thoman, 1964, Wollman et al., 1956). In the electron microscope, the only visible manifestation of the F system has been its pilus (Folkhard et al., 1979). The pilus of conjugative T4S systems is not only an essential cylindrical conduit for conjugating DNAs, but also is the first point of entry for many phages, which attach to T4S systems pili before injecting their DNA or RNA into bacterial cells (Arutyunov and Frost, 2013). In this era of widespread antibiotic resistance and regained interest in phage therapy to combat bacterial infections, it is essential to understand phage-pilus interactions. A crucial step toward elucidating this interaction is the determination of its structural basis. However, while rapid progress in the structural biology of phages has been made, no atomic resolution details are available for T4S system pili. Previous studies have provided some confusing insights into the helical parameters of the F pilus and were of insufficient resolution to derive an atomic model (Folkhard et al., 1979, Marvin and Folkhard, 1986, Wang et al., 2009). Here, we present structural details for two F family pilus types, the pED208 and F pili, derived from 3.6 Å and 5.0 Å resolution cryoelectron microscopy (cryo-EM) maps, respectively. These structures provide unprecedented details of conjugative pilus architecture and function.

## Results and Discussion

### Pilus Production and Structure Determination

**Figure 1:**
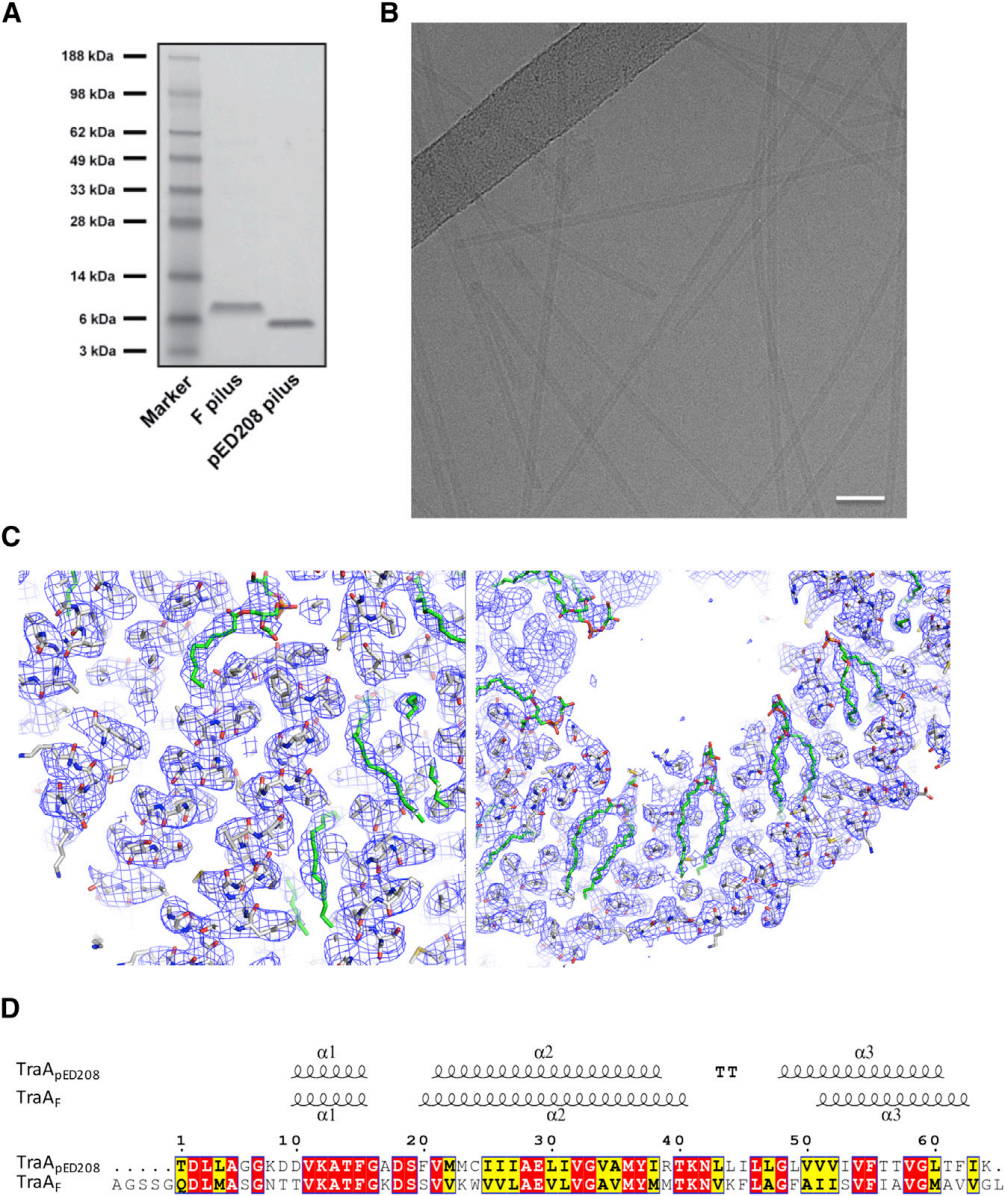
Purification of the pED208 and F Pili and Cryoelectron Microscopy of the pED208 Pilus (A) SDS-PAGE of purified pED208 and F pili. (B) Electron micrograph of the pED208 pilus. The scale bar represents 40 nm. (C) Details of two representative regions of the experimentally derived density for the pED208 pilus. The electron density map contoured at a 1.5σ level is shown in chicken wire representation colored in blue. The pED208 TraA model is in stick representation with atoms color-coded light gray, blue, and red for carbons, nitrogens, and oxygens, respectively. The lipid model is PG in stick representation color-coded green, yellow, blue, and red for carbon, phosphorus, nitrogen, and oxygen, respectively. For clarity, two views are provided: one in which the protein structure is clearly apparent (left) and the other where the lipid structure is clearly apparent (right). (D) Sequence alignment of pED208 and F TraA. Identical and similar amino acids are boxed in red and yellow, respectively. Secondary structures of pED208 and F TraA are shown above the sequence alignment.

**Figure S1:**
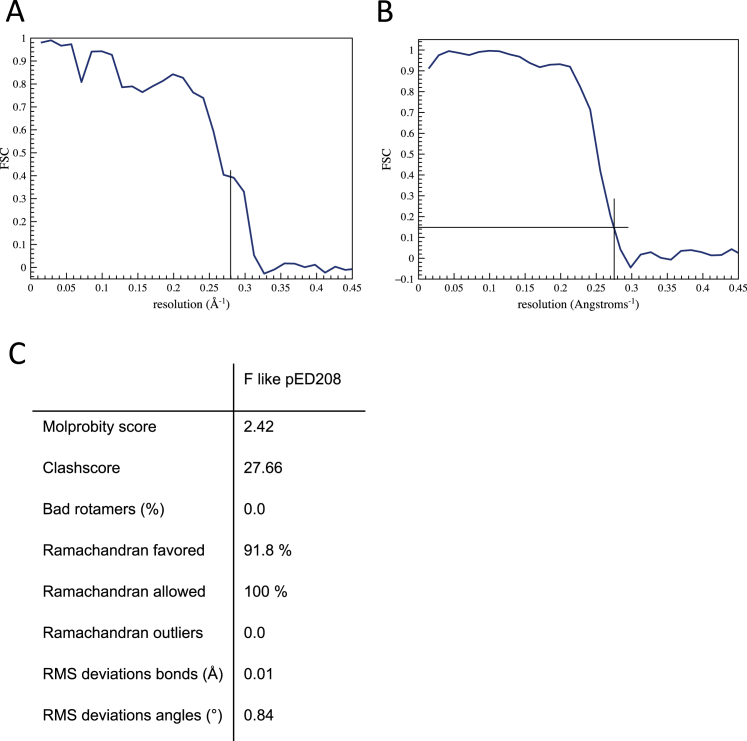
Resolution and Model Statistics for the pED208 Pilus Structure, Related to Figure 1 (A and B) (A) Resolution of pED208, as derived from Fourier Shell Correlation (FCS) calculation between the stereochemically refined atomic model and the map. This yields an FSC = 0.4 at a resolution of 3.6 Å. Since the traditional FSC between two half-maps is not a measure of resolution, but rather of self-consistency (Egelman, 2014), the model:map FSC provides a better measure of actual resolution. We show, however, in (B) an FSC between two independent half-maps, which yields a resolution of 3.6 Å at FSC = 0.143, consistent with the model:map FSC in (A). In addition, and more importantly, the visual appearance of the map is also consistent with a resolution of 3.6 Å. (C) Model statistics from MolProbity.

**Figure S2:**
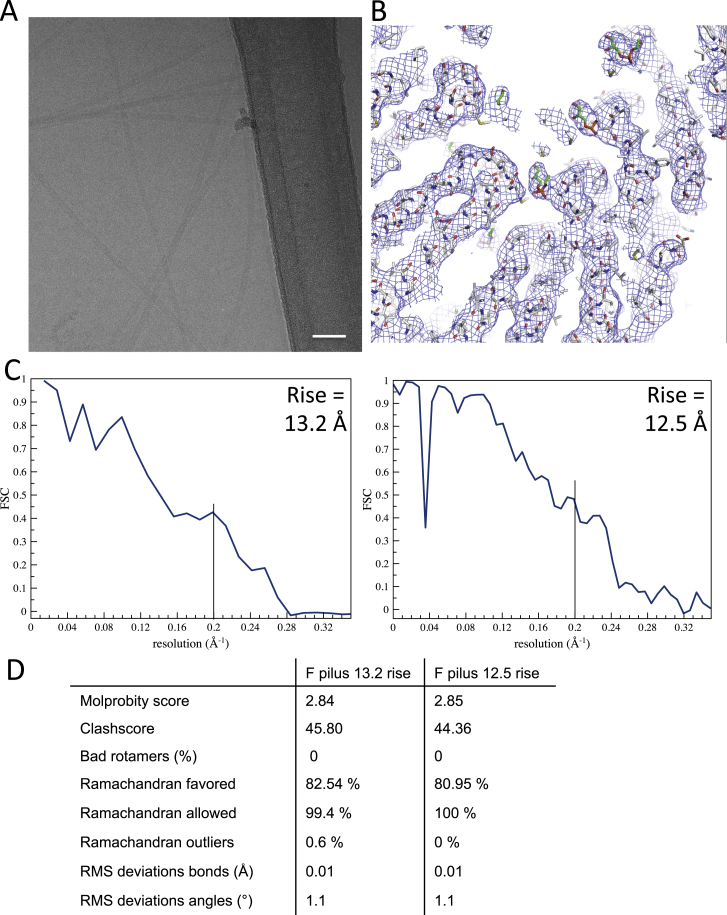
Structure of the F Pilus, Related to Figure 1 (A) Electron micrograph of the F pilus. The scale bar represents 40 nm. (B) The 5Å resolution electron density map of the 12.5Å rise F pilus filament. The region shown is the same as the one presented in Figure 1C. The map is contoured at the same level. (C) Resolution estimated for the two F pilus models, using a map:model FSC. Both show a resolution of 5 Å at FSC = 0.4. (D) Refinement statistics for the two F pilus models.

The F and the F-like pED208 plasmids are two plasmids that encode their own T4S systems and thus produce their own pili. The F and pED208 pili were produced in vivo and were purified as described in STAR Methods (Figure 1A). The pili were applied to grids and were vitrified for cryo-EM analysis (Figure 1B). Data collection and structure determination proceeded as described in STAR Methods. For pED208, a 3.6 Å resolution map was generated (Figures 1C, S1A, and S1B) in which α-helical secondary structures, as well as most side chains, were clearly visible (Figure 1C) and in which a model for the pED208 pilin, TraA, could be readily built and refined with excellent stereochemistry (Figure S1C). During the process of helical reconstruction, as the resolution was increased, an additional separate density became clearly visible and readily interpretable as a phospholipid (Figure 1C). For the F pilus, it became apparent during the process of helical reconstruction that two populations of filaments were present, differing slightly in the rise between subunits (see below). Near-atomic resolution for the F pilus was not achieved, presumably because the F pilus might not be as ordered as the pED208 pilus. Instead, two 5.0 Å resolution electron density maps were generated for the two F pilus populations. These maps clearly showed helical secondary structures in which models of the F pilin, TraA, could be built and refined (Figures S2A–S2D).

### General Architecture of the pED208 and F Pili

**Figure 2:**
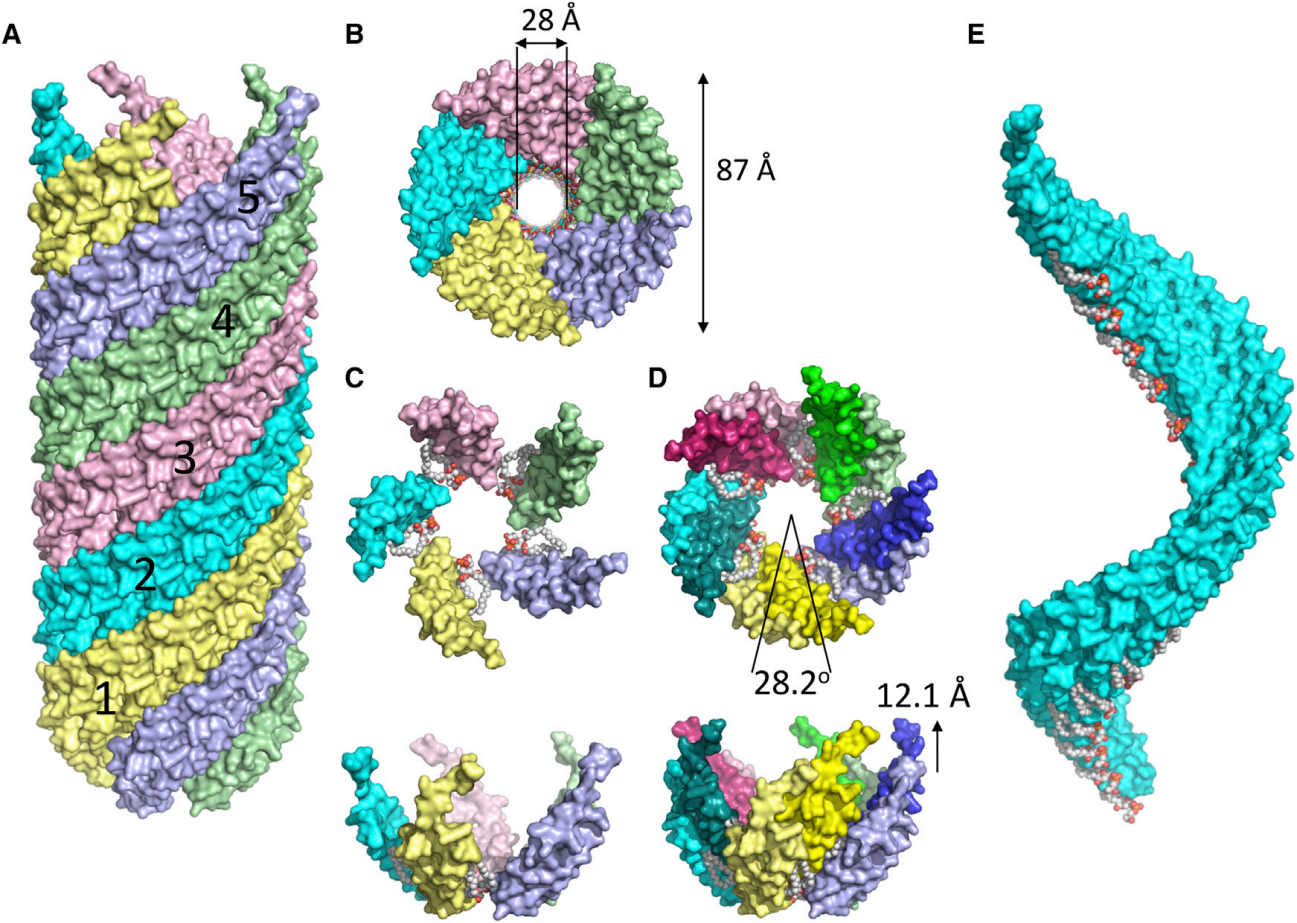
Overall Architecture of the pED208 Pilus (A) Side view of the pED208 pilus structure. The structure is in surface representation. It consists of a five-start helical assembly. Each of the five helical strands is shown in a different color and is labeled 1–5. Each helical strand consists of 12.8 subunits per helical turn. Thirteen subunits are shown and named A–M from bottom to top. Although the overall orientation of the pilus relative to the membrane is not known, we hypothesize that the membrane-proximal end of the pilus is at the bottom. This is based on the fact that the α2-α3 loop is known to be cytoplasmic when the pilus subunit is inserted in the membrane (Paiva et al., 1992). Since, in the structure of TraA determined here, the loop is orientated down within the pilus, this would also position the membrane-proximal end of the pilus down. (B) Bottom view of the pED208 pilus structure. Representation is as in (A), except that the lipid head group atoms (represented as spheres color-coded white, yellow, and red for carbon, phosphorus, and oxygen atoms, respectively) are visible inside the lumen of the pilus. The external and internal dimensions of the pilus are reported. (C) The pentamer unit of the pED208 pilus. Each subunit and lipid is in surface and sphere representation, respectively. This figure was generated using the TraA molecule labeled I in each helical strand. Color-coding is as in (A) and (B). Top, top view. Bottom, side view. (D) Two adjacent pentamer units of the pED208 pilus structure. The pentamer unit at the base is as in (C) (made from the TraA molecules named I, i.e., the ninth subunit in each helical strand) while the pentamer unit above (made from the TraA molecules named J, i.e., the tenth subunit in each helical strand) is shown in similar but stronger colors. The lipids are as in (A). The angle and rise between equivalent subunits in consecutive pentamer stacks are reported. Top, top view. Bottom, side view. (E) The PG array in the context of the pilus strand. Pilus strand 2 of (A) is shown together with bound PG. Representation of the strand is as in (A), while representation of the PG is as in (C). This image clearly shows the continuous PG array along the pilus strand.

The structures of the pED208 and F pilus are very similar, with overall dimensions of 87 Å in diameter and an internal lumen of 28 Å in diameter (Figures 2A and 2B). They can be described in two equivalent ways, as illustrated in Figure 2: (1) as five-start helical filaments (Figures 2A and 2B where the five helical strands are color-coded differently), or (2) as pentamer layers stacked on top of each other, each layer related to the one below or above by a rotation angle and rise (Figures 2C and 2D). For pED208, the angle between adjacent subunits is 28.2° and the rise is 12.1 Å (Figure 2D), while for the two F pilus structures, the helical parameters were 27.9° with a rise of 13.2 Å and 28.1° with a rise of 12.5 Å, respectively. Thus, the general pED208 and F pilus architectures can be considered virtually identical. Previous published work has reported different helical parameters for the F pilus (Wang et al., 2009), but those were incorrect due to the low resolution achieved. At the near-atomic resolution reported here, there can be no ambiguities as to the assessment of the symmetry and, therefore, the parameters reported here are definitive (Egelman, 2014).

The amino acid sequences of both the orthologous pED208 and F pilus TraA pilin are similar, except for the F pilin N terminus, which is 4 amino acids (aa) longer (Figure 1D). This longer N terminus could not be traced in the F pilus density maps and thus must be disordered. When seen as a five-start helical filament, the pilus displays five helical strands (labeled 1–5 in Figure 2A), each made of ∼12.8 TraA pilins per turn. Since the structures of the pED208 and F pili are very similar but much higher resolution was achieved for the pED208 system, we will focus all subsequent description of pilus architecture and pilin structure on this pilus type, pointing to notable differences with the F system when required.

### Structure of the TraA Pilin

**Figure 3:**
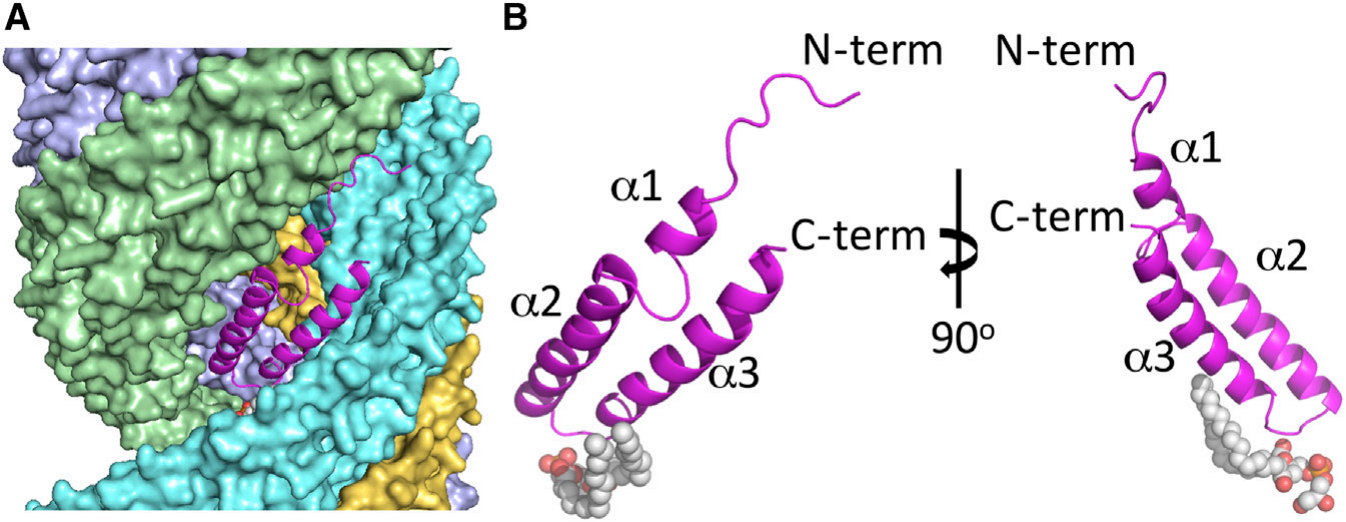
Structure of pED208 TraA (A) Location of the subunit shown in (B) within the pilus. For clarity, and in order to maintain the same orientation throughout, a subunit (subunit I in helical strand 3) was chosen arbitrarily as the reference subunit. (B) Structure of the TraA-phospholipid complex. TraA is in ribbon representation with the N, C terminus, and secondary structures labeled. The lipid is in sphere representation color-coded as in Figure 2C. Left, orientation of TraA is as in (A). Right, orientation of TraA is 90 degrees away from orientation at left.

**Figure S3:**
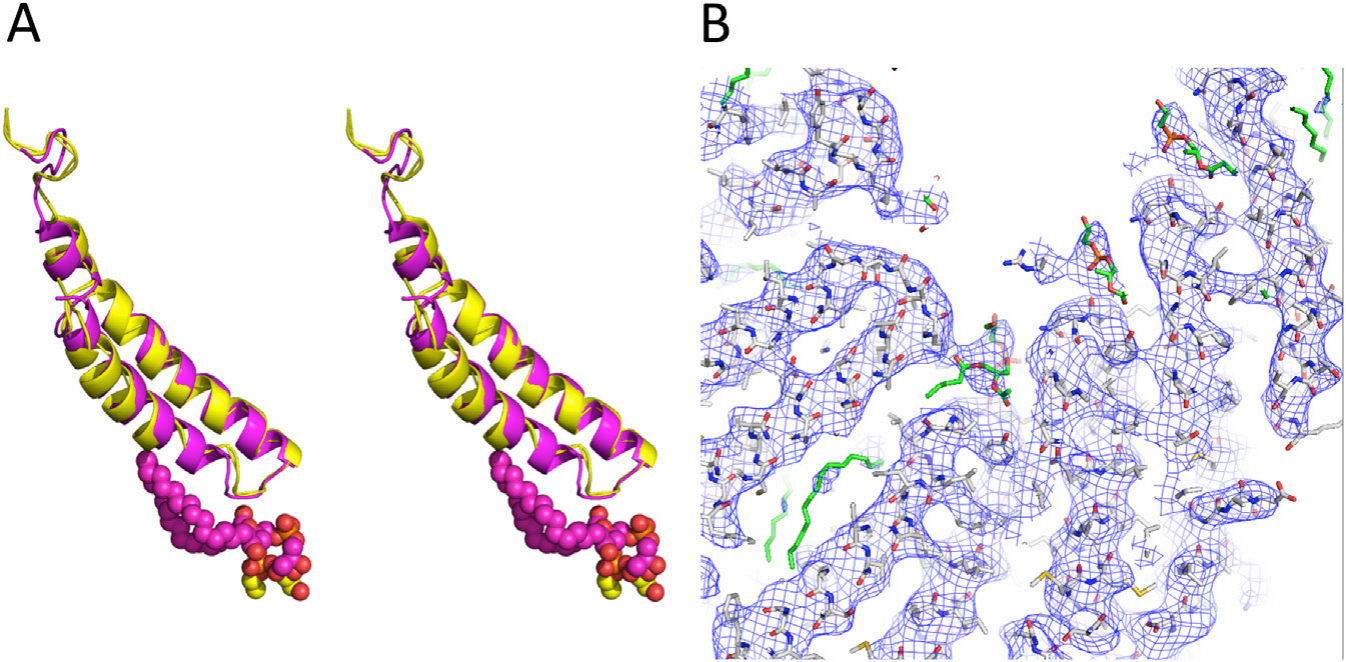
Structural Comparison of F and pED208 Pilus Subunit and Lipid Structures, Related to Figures 2 and 3 (A) Superposition of the F and pED208 TraA structures. The proteins and lipids are in ribbon and sphere representations, respectively. Color-coding for the protein is magenta and yellow for pED208 and F TraA (rise 13.2 Å), respectively. For the lipid, color-coding is by atom type with oxygen, nitrogen, and phosphorus atoms in red, blue, and pink, respectively, and carbon in magenta and yellow for the lipid bound to pED208 or F TraA, respectively. (B) Representative region of the pED208 pili density map at 5 Å resolution around the lipid-binding site. This panel needs to be compared to the 5 Å resolution map for the F pili structure shown in Figure S2B. As can be inferred from such comparison, at the resolution of 5 Å, the densities for the lipid and protein are very similar. For the pED208 pili structure, the resolution could be extended to 3.6 Å and the density for this region resolves unambiguously the structure of a phospholipid. Map representation and models are as in Figure 1C.

The pED208 TraA pilin is a 64-residue protein, 63 of which (residues 2–64) were clearly defined in the electron density. TraA folds into an all-α-helical structure, containing three α helices (α1–3) (Figures 3A and 3B). A 9-residue N terminus extending out is followed by α1, a short helix, which forms a two-helix bundle with the C-terminal end of α3. A 5-residue loop between α1 and α2 (the α1-α2 loop) protrudes and folds back into the core of the pilin structure to be followed by α2, a longer helix that forms an extended two-helix bundle with the N-terminal part of α3. Thus, α3 itself interacts with both α1 and α2. TraA orientation within the pilus is such that the loop between α2 and α3 is located within the lumen of the pilus, while the N- and C-terminal ends are located on the outside of the filament. This is consistent with previous results suggesting that the N- and C-terminal regions of the F TraA pilin are accessible for phage attachment and thus must be located on the outside (Frost and Paranchych, 1988). It is also consistent with prior suggestions that the α2-α3 loop might be involved in contacting the DNA as it passes through the pilus (Paiva et al., 1992, Silverman, 1997). Superposition of the structures of pED208 and F TraA (Figure S3A) reveals very similar overall structures (root-mean-squared deviation in Cα positions between pED208 and F [13.2 Å rise] of 1.4 Å and between pED208 and F [12.5 Å rise] of 1.5 Å) with slightly different boundaries for secondary structures (Figure 1D). However, the F TraA structure was solved at a lower resolution, and thus, whether these minor differences are significant remains unclear. Also, the TraA pilin structures in the two F pilus forms are virtually identical (root-mean-squared deviation in Cα positions of 0.7 Å). Thus, with the structures and helical parameters of the two F pilus forms being so similar, functional differences between them are unlikely to arise but cannot be excluded.

### The F and F-like Pili Are Helical Assemblies of Stoichiometric Protein-Phospholipid Units

**Figure 4:**
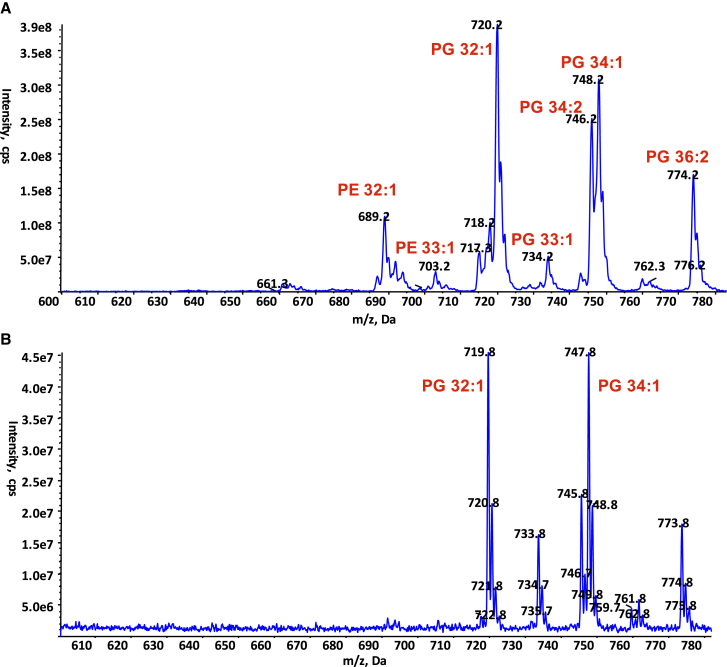
MS Analysis of the Lipids Extracted from pED208 Pili (A) Negative ion mode survey scan (600–780 mass/charge ratio [m/z]) of lipid extracts from whole-cell membranes. (B) Negative ion mode survey scan (600–780 m/z) of lipid extracts from purified pili pre-treated with PLA2. In all cases, phospholipids’ identity was confirmed by daughter fragmentation and reported here.

Early on during the process of helical reconstruction of the pED208 pilus, additional unconnected density in the vicinity of the base of helix α3 became visible (see Figure S3B showing density map at 5Å resolution). As resolution increased, clear density resolved the head group and acyl chains of a phospholipid stoichiometrically interacting with the pilin (Figures 1C and 2E). This finding was confirmed in experiments (Figure 4) in which the purified pED208 pili were first treated with phospholipase 2 (PLA2) and the remaining bound lipids subsequently extracted and analyzed by mass spectrometry (MS). Two main species bound to the pilin were identified by daughter ion fragmentation as phosphatidylglycerol (PG) species, PG 32:1(16:0, 16:1) and PG 34:1 (16:0, 18:1) (Figure 4). These are also major PG species in the whole-cell membrane (Figure 4A). However, selectivity is observed, as there is no presence of the other two major phospholipid classes, phosphatidylethanolamine (PE) (compare Figure 4B with Figure 4A) and cardiolipin (data not shown), in the PLA2-treated pili extracts. Moreover, while the total PG pool only accounts for ∼19% of the total phospholipid content of the *E. coli* membrane, the two major PG species identified in the pilus account for 72% of the lipid content of the pilus (Figure 4A).

**Figure S4:**
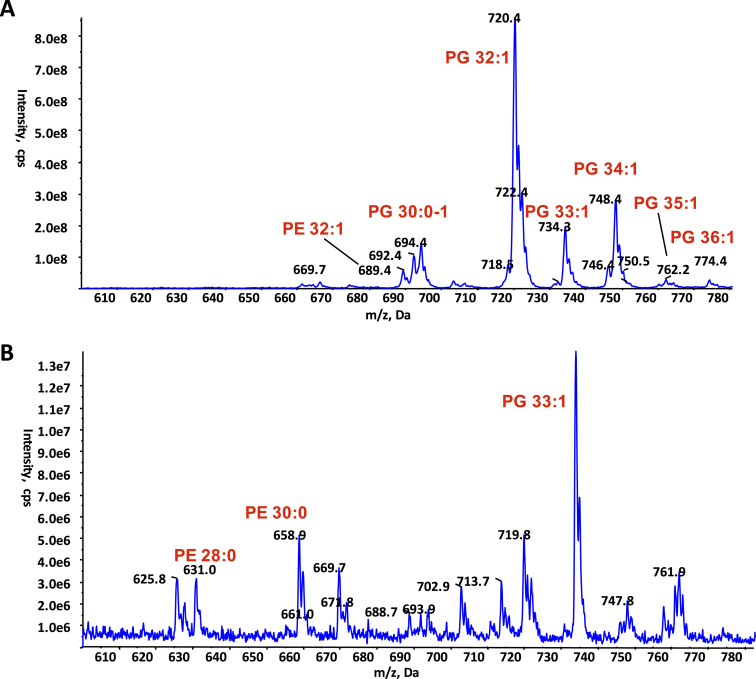
MS Analysis of the Lipids Extracted from F Pili, Related to Figure 4 (A) Negative ion mode survey scan (600–780 m/z) of lipid extracts from whole cell membranes. (B) Negative ion mode survey scan (600–780 m/z) of lipid extracts from purified pili treated with PLA2. In all cases, phospholipids identity was confirmed by daughter fragmentation.

For the F pilus, additional density was also observed at the same location, and its shape was similar to the electron density observed for the pED208 at 5Å resolution (Figures S2B and S3B show the same region in the 5Å maps of F and pED208, respectively), suggesting that a phospholipid molecule is also bound stoichiometrically to the F TraA protein. Indeed, the presence of phospholipid in the F pilus was also confirmed by MS (Figure S4); however, this time a PG species, PG 33:1 (16:0, ΔC17:0), was the major phospholipid observed in the F pilus after PLA2 treatment (Figure S4B). This PG species is only a minor PG species of the total cell lipid extract (Figure S4A). Thus, selective binding of PG to pilins occurs in both F and pED208 pili, and thus, the F family of pili are polymers of a selective and stoichiometric protein-PG complex unit.

### The Pilin-Lipid Interaction Network in the pED208 Pilus

**Figure 5:**
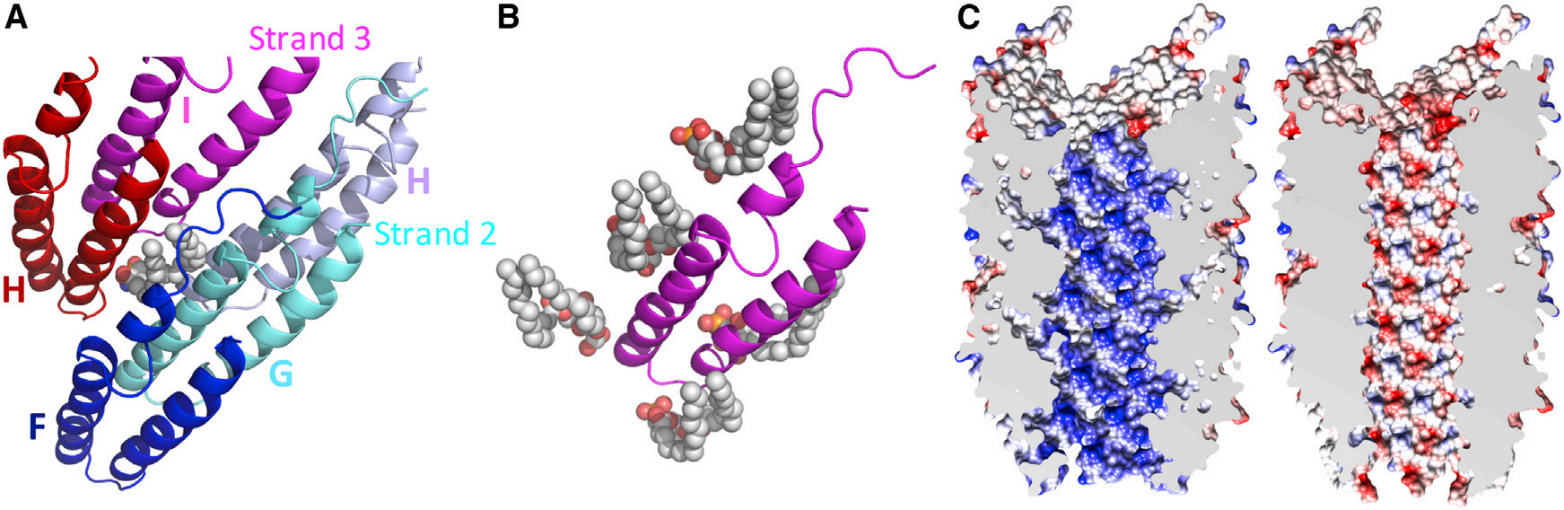
Overview of Lipid-Protein Interactions (A) Each lipid interacts with five adjacent TraA subunits. TraA subunits and phospholipids are shown in ribbon and sphere representation, respectively. Orientation of the reference magenta subunit (labeled I) is as in the left panel in Figure 3B. The lipid molecule is at the interface between subunits in strands 2 and 3. (B) Each TraA subunit interacts with five phospholipid molecules. Representation and color-coding are as in Figure 3B. (C) Electrostatic potential of the pilus lumen calculated without (left) or with (right) the phospholipids. The lipids were included in the model as described in STAR Methods. Electrostatic potential and surfaces were calculated using CHIMERA.

**Figure 6:**
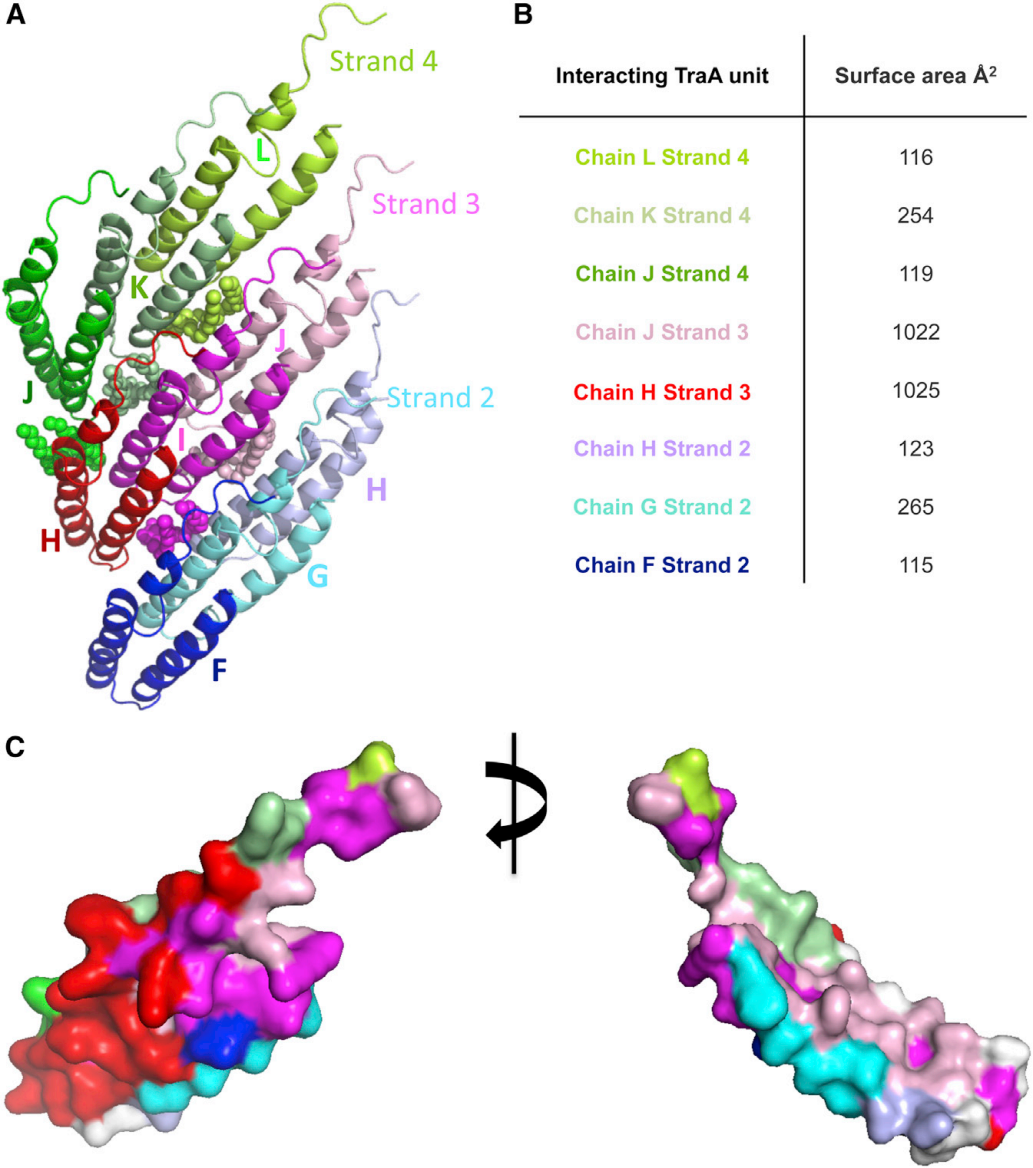
Protein-Protein Interaction Networks in the pED208 Pilus (A) Each subunit interacts with eight others within the pilus. All subunits interacting with the reference subunit in magenta (subunit I in strand 3) are shown, as well as their associated phospholipid. All subunits and lipids are in ribbon representation color-coded various shades of green, red, and cyan for subunits in helical strands 4, 3, and 2 (as defined in Figure 2A), respectively. Subunits are labeled J, K, and L in strand 4; H, I, and J in strand 3; and F, G, and H in strand 2. In this nomenclature, each of the 13 subunits in each helical array was labeled A–M, with subunit A at the bottom of the pilus structure model. (B) Surface area buried in subunit-subunit interactions. The reference subunit used in these calculations is in magenta in (A). Color-coding is as in (A): for example, number in cyan indicates surface area buried between subunits in magenta and cyan in (A). (C) Mapping of subunit-subunit interactions onto the reference subunit. The reference subunit is in magenta in (A). Interactions made between the reference subunit and the subunit in red in (A) are mapped onto the reference subunit surface by color-coding its surface in red. The same is carried out for all other subunits shown in (A). The result is the mapping of interactions that each subunit makes with the reference subunit. Interactions with lipids are mapped in white.

**Figure S5:**
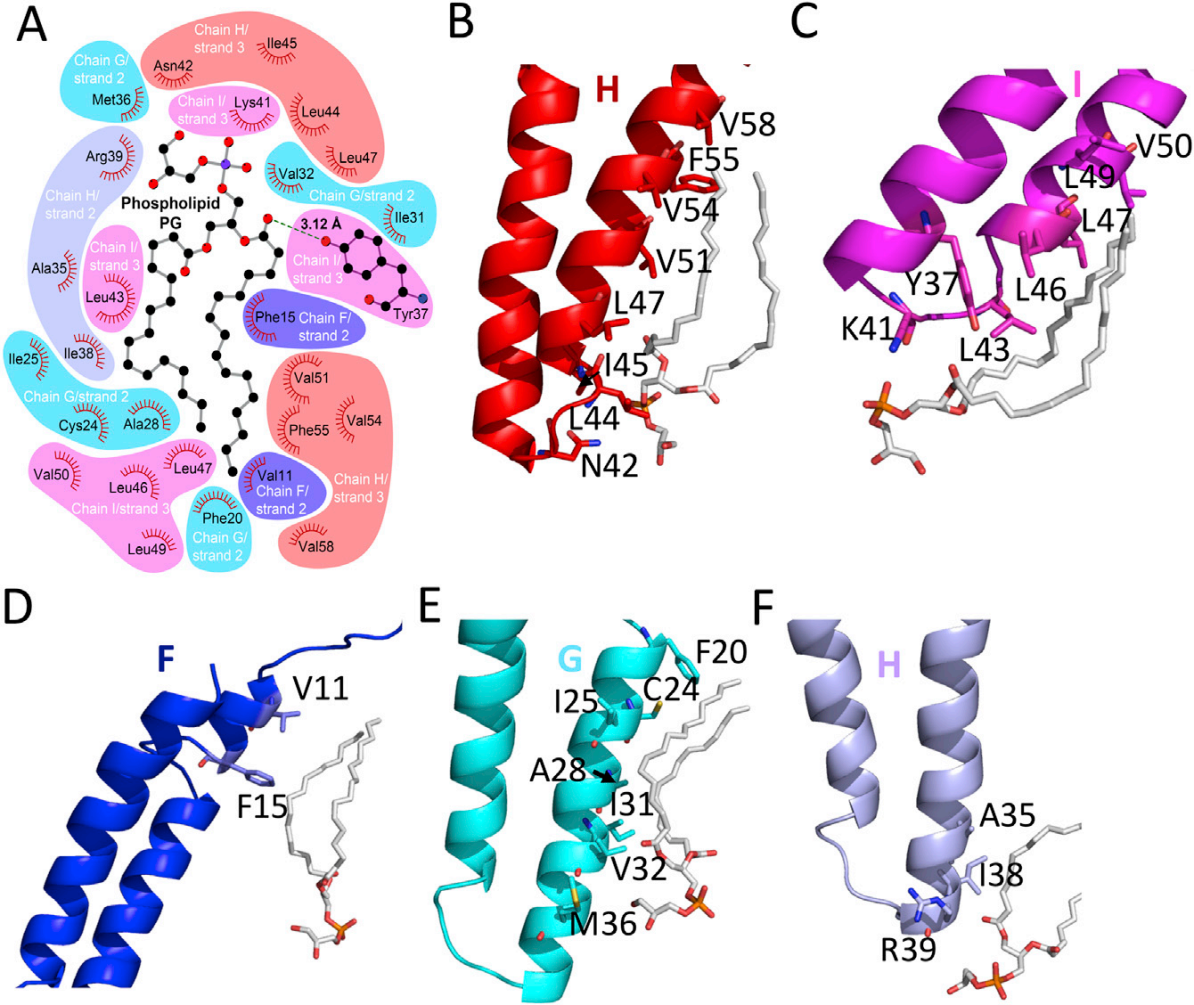
Details of pED208 TraA-phospholipid Interactions, Related to Figure 5 (A) Interaction diagram between one phospholipid and five adjacent TraA subunits. The interactions are between the phospholipid and the subunits shown in Figure 5A, using the same color-coding and naming for TraA subunits. This diagram was created using LIGPLOT (Laskowski and Swindells, 2011). (B–F) Detailed side-chain interactions with each subunit. TraA subunits are shown in ribbon color-coded and labeled as in Figure 5A. Interacting residues are shown in stick representation color-coded with blue and red indicating nitrogen and oxygen atoms, while the carbon atoms are color-coded as in the ribbon. PG lipid is in stick representation with atoms color-coded white, blue, red, and orange for carbon, nitrogen, oxygen, and phosphorus, respectively. (B) Side chains involved in the interaction of the PG bound to chain I of strand 3 with TraA chain H in strand 3. The hydrophobic chain of PG interacts with side chains of hydrophobic residues Val58, Phe55, Val54, Val51, Leu47, and Ile45. The head group of the phospholipid interacts with Leu44 and Asn42 through hydrophobic and hydrogen bond interaction, respectively. (C) Side chains involved in the interaction of the PG bound to chain I of strand 3 with TraA chain I in strand 3. The hydrophobic chain of PG interacts with Val50, Leu49, Leu47, Leu46, and Leu43. Two hydrogen bond interactions were observed between the head group of PG and the main chain carbonyl group of Lys41 and between PG *sn-*2 oxygen with the hydroxyl group of the Tyr37 side chain. (D) Side chains involved in the interaction of the PG bound to chain I of strand 3 with TraA chain F in strand 2. The hydrophobic chain of PG interacts with the side chains of Val11 and Phe15. (E) Side chains involved in the interaction of the PG bound to chain I of strand 3 with TraA chain G in strand 2. The hydrophobic tail of PG interacts with Phe20, Cys24, Ile25, Ala28, Ile31, and Val32 with the PG head group closer in proximity to Met36. (F) Side chains involved in the interaction of the PG bound to chain I of strand 3 with TraA chain H in strand 2. The hydrophobic tail of PG interacts with Ala35 and Ile38, while the Arg39 main chain carbonyl oxygen makes a hydrogen bond with the PG head group.

The pilus is held together by interactions not only between pilin subunits, but also between lipids and subunits (Figures 5 and 6). Each lipid molecule makes extensive contacts with five surrounding TraA subunits (Figure 5A), while each TraA subunit interacts with five lipid molecules (Figure 5B). Overall, 70.3% of the lipid’s surface is buried (769 Å^2^ against 1094 Å^2^ total), while 16.7% of each subunit is involved in contact with phospholipids (912 Å^2^ against 5449 Å^2^ total). In the lipid, only the head groups are solvent exposed and directed to the lumen of the pilus (Figure 5C). The acyl chains are entirely buried between subunits. Details of residue-specific pilin-lipid interactions are described in Figure S5. These involve primarily hydrophobic residues interacting with the acyl chains. Only very few but significant contacts with the phospholipid head group are observed (between the phosphate and K41 and Y37, for example).

The composition of the lumen is unique in being lined not only with residues from the α2-α3 loop, but also with the lipid head groups. In the F pilus, this loop, referred to as the “KNVK” loop, was hypothesized to form a contact with ssDNA during conjugative transfer (Paiva et al., 1992). The structures presented here locate this loop to the lumen of the pilus, suggesting that indeed the pilus serves as a conduit for ssDNA transfer. Remarkably, integral to the lining of the lumen is the stoichiometric inclusion of phospholipid head groups. To gain further insight into the potential impact that inclusion of PG head groups within the lumen lining might have, the electrostatic potential of the lumen was calculated with or without PG (Figure 5C). Inclusion of PG has a profound impact on the electrostatic potential of the pilus lumen: without PG, it is overwhelmingly positive, while with PG, it is moderately electronegative. By generating a conduit with a moderately negative inner surface, phospholipids may facilitate transport of the negatively charged ssDNA substrate.

### Mutational Studies of Lipid-Interacting Residues Confirm the Importance of Lipid Binding in Preserving the Integrity of the Pilus

**Figure 7:**
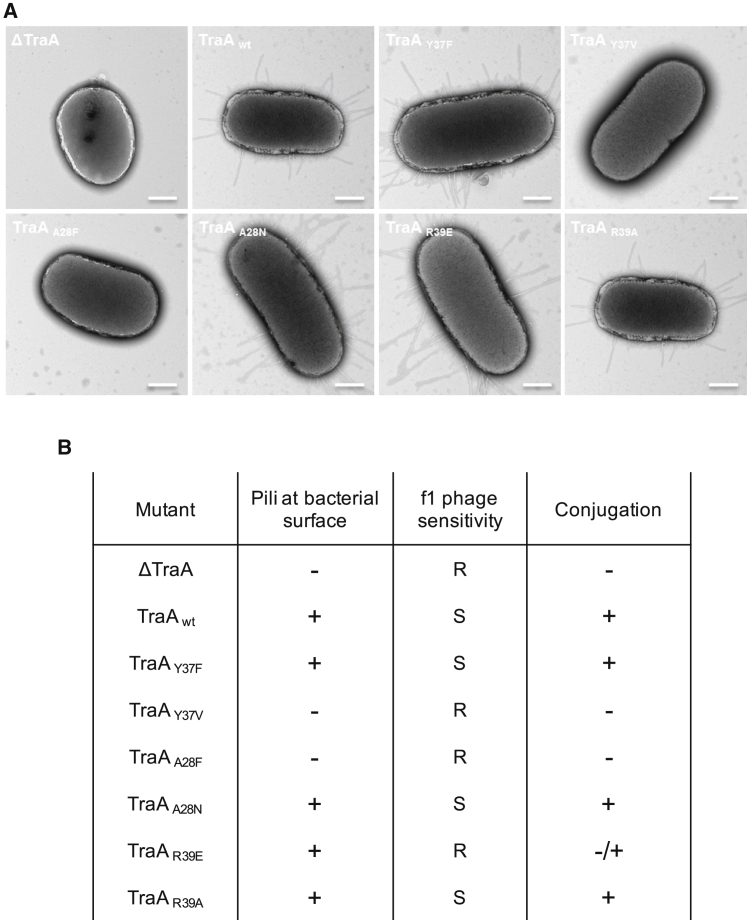
Effect of pED208 TraA Mutations on Pilus Formation and Function (A) NS-EM micrographs of JE2571 cells harboring a *traA* deletion (pED208_ΔTraA) in the pED208 plasmid and complemented in *trans* with wild-type (WT) or point mutated TraA pilins. See main text for mutant description. The scale bar represents 500 nm. (B) Summary of the effect of TraA single point mutations in pili formation, f1 phage sensitivity, and conjugation efficiency. Minus sign (−), no pili or conjugation reduced by 10^−4^-fold; plus sign (+), pili or conjugation observed at WT level; (−/+), intermediate (10-fold decrease compared to WT) level of conjugation; (R) resistance or (S) sensitivity to phage f1.

The F TraA pilus subunit has been subjected to extensive mutagenesis (Frost and Paranchych, 1988, Manchak et al., 2002). All mutations observed to affect pilus biogenesis locate to protein-protein interfaces, while mutations affecting conjugation and phage attachment locate to either the lumen or the periphery of the pilus. Thus, the structure presented here provides the structural basis for all published F pilus mutations. However, the PG-binding site was never targeted for mutation, as it was unknown. Three residues in the interface between PG and pED208 TraA were thus chosen for mutational analysis (the location of these mutants is shown in Figure S5): A28 makes hydrophobic interactions with the acyl chain of PG and was mutated to F or N (TraA_A28F_ and TraA_A28N_); Y37 makes an H-bond to the carbonyl oxygen of the *sn-*2 fatty acid and was mutated to F or V (TraA_Y37F_ and TraA_Y37V_); finally, R39, an important residue whose amide nitrogen also contacts the carbonyl oxygen of the *sn-*2 fatty acid and whose side chain not only makes up some of the lumen lining, but also contacts other adjacent TraA molecules, was mutated to E or A (TraA_R39E_ and TraA_R39A_). Pilus biogenesis and function were assessed using negative-stain EM for observation of pilus production at the bacterial cell surface or using conjugation and filamentous phage f1 infection for observation of pilus function (Figure 7; see details in STAR Methods). Mutating A28 to the bulky residue F is expected to create severe steric clashes and to be disruptive of pilus biogenesis, and this is precisely what is observed. Substitution to N at this position is less drastic and results in minimal disruption of pilus function. Mutation of Y37 to F is ineffective, demonstrating that the hydrogen bond between Y37 and the lipid is a minor element in PG-TraA interaction but the aromatic side chain is critical, as mutation to V impairs pilus formation and function. As expected, mutation of R39 to A preserves the structural integrity of the pilus, since the main interaction of R39 with the lipid is through its main-chain amide nitrogen. However, inverting the charge at this position appears to decouple conjugation from phage infection: indeed, R39E does not affect pilus biogenesis and has only a small impact on conjugation but completely abrogates phage infection. During conjugation, the pilus is known to serve as an export conduit for a mixed nucleo-protein complex consisting of the relaxase protein covalently bound to ssDNA, itself possibly coated with single-strand DNA-binding proteins (Ilangovan et al., 2015); in contrast, during phage DNA import, a naked electro-negative nucleic acid passes through the pilus lumen (Caro and Schnös, 1966, Crawford and Gesteland, 1964). An R39E mutation would strongly increase the electro-negative potential within the lumen of the pilus, thereby giving rise to a strong repulsive force that would prevent the negatively charged DNA of the phage from entering the pilus conduit. This would not be the case with the more electrostatically neutral protein-DNA complex that serves as a substrate during conjugation.

### The Pilin-Pilin Interaction Network in the pED208 Pilus

**Figure S6:**
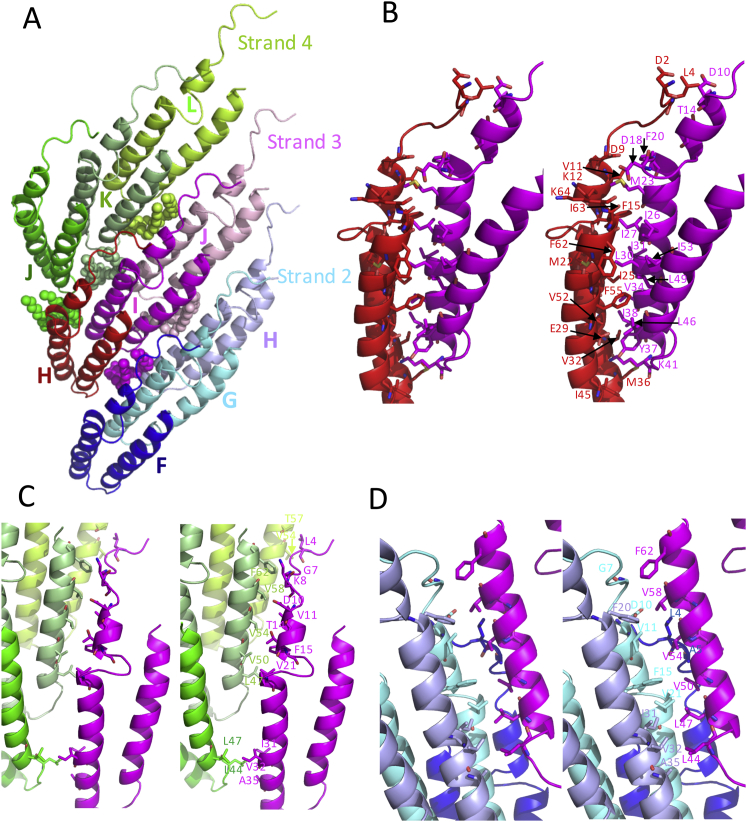
Details of TraA-TraA Interactions, Related to Figure 6 (A) Same as Figure 6A, repeated here for clarity. (B) Interactions between chain I and chain H of strand 3 represent the largest set of interactions between two TraA monomers within the pED208 pilus. These two TraA pilin subunits share a large hydrophobic interaction surface involving numerous residues in α2 and α3. From the bottom of the panel, closer to the lumen, the side chains of Met36, Val32, and Ile45 in chain H make contact with Ile38 of chain I. Also, the side chains of residues Lys41 in chain I and Glu29 in chain H make contact through a salt bridge interaction. Further, a central-core-buried hydrophobic surface constituted by the side chains of Val52, Phe55, Phe62, Ile63, Ile25, Met22, Phe15, and Val11 in chain H and Tyr37, Leu46, Val34, Leu49, Ile53, Ile26, Ile27, Leu30, Ile31, Met23, and Phe20 in chain I stabilizes the interaction core between the two TraA monomers. Residues Asp9, Lys12, and Ile63 of chain H are in close proximity to Met23 of chain I. Lys64 at the C-terminal end of chain H interacts with the side chain of Asp18 in chain I through a hydrogen bond. At the N-terminal end of the chains, Leu4 of chain H makes contact with Thr14 of chain I. Asp2 of chain H and Asp10 of chain I form a stabilizing hydrogen bond through their main chain groups. (C) Interactions between chain I in strand 3 and chains J, K, and L in strand 4. Starting from the top of the panel, residue Leu4 in chain I of strand 3 interacts with Thr57 and Val54 of chain L in strand 4. Residues Gly7, Lys8, Asp10, Val11, Thr14, Phe15, and Val21 of chain I in strand 3 interact with chain K of strand 4 through residues Phe62, Val58, Val54, Val50, and Leu47. Finally, Ile31, Val32, and Ala35 in chain I of strand 3 are in proximity to Leu47 and Leu44 of chain J in strand 4, stabilizing the two chains through hydrophobic interactions. (D) Interaction between chain I of strand 3 with chains F, J, and H of strand 2. Starting from top of the panel, residue Phe62 in chain I of strand 3 is in proximity to Gly7 of chain G in strand 2. Val58 of chain I in strand 3 is in proximity to Val11 and Asp10 of chain G in strand 2, Phe20 of chain H in strand 2, and Leu4 of chain F in strand 2. Val54 of chain I in strand 3 is surrounded by hydrophobic residues Val11, Phe15 of chain G in strand 2, and Leu4 and Ala5 of chain F in strand 2. The residue Val50 of chain I in strand 3 interacts with Phe15 and Val21 of chain G in strand 2 and also with Ala5 of chain F in strand 2. Leu47 in chain I of strand 3 interacts with Ile31 of chain H in strand 2 and Val21 of chain G in strand 2. Finally, Leu44 of chain I in strand 3 is in proximity to Val32 and Ala35 of chain H in strand 2. All figures and supplementary figures showing structural data were generated using PYMOL (Molecular Graphics System, Version 1.8 Schrödinger, LLC).

Each TraA molecule makes contact with eight adjacent subunits (Figure 6A). The subunit-subunit interaction networks involved adjacent subunits in the same helical strand (for example, in Figure 6A, subunit labeled I in helical strand 3 makes contacts with the previous and subsequent subunit within the strand, labeled H and J, respectively), but also with three subunits in the strand above (helical strand 4, subunits labeled J, K, and L) and below (helical strand 2, subunits labeled F, G, H; see notation of pilus subunits in legend to Figure 6). One consequence of this arrangement is that the entire length of each subunit is involved in contact with other subunits either in the same helical strand or in the strands above and below. The surface area buried in protein-protein interactions between a reference subunit (subunit I in strand 3) is reported in Figure 6B: overall 3,043 Å^2^ of subunit surface is buried in protein-protein contacts. Once contacts with the lipid molecules are taken into account, 72% of the subunit surface is buried (Figure 6C). The only solvent-accessible surfaces are at the periphery, either facing outward for phage attachment or facing inward toward the lumen for DNA transport. Details of subunit-subunit interactions are shown in Figure S6.

### Conclusions

The structure of the F pilus reveals a protein-phospholipid complex as the primary unit from which the pilus is assembled. Prior to assembly, each TraA molecule is embedded in the inner membrane and then extracted from the membrane during pilus biogenesis (Paiva et al., 1992). The stoichiometric presence of phospholipid within the pilus demonstrates that, as pilins are extracted from the membrane, each remains associated with one phospholipid molecule. Lipids have been observed bound to proteins but often as a result of unspecific binding. Only lipid metabolizing enzymes form stoichiometric complexes with their substrates. Thus, to our knowledge, our observation of a lipid bound stoichiometrically to a protein polymer is unprecedented. Moreover, the lipid composition of the pilus is different from that of the membrane, suggesting preferential binding of TraA to a subset of phospholipids. These observations thus have important biological implications: (1) the presence of lipid within the pilus structure might facilitate pilus insertion into host membranes so as to be able to deliver substrates to recipient host cells; (2) the presence of lipid might also facilitate re-insertion of pilus subunits within the inner membrane during pilus retraction/depolymerization; and (3) differential selectivity among conjugative pili for specific lipid species might increase the range of substrate selectivity. An essential aspect of pilus function is indeed its ability to enter successive cycles of growth and retraction (Clarke et al., 2008), a function that is likely essential for conjugation but also that has been shown to be necessary during infection to bring phages closer to the membrane (Riechmann and Holliger, 1997). One can reasonably hypothesize that stripping off all bound lipids from TraA would have an energetic cost, as would the requirement of partitioning a lipid-free TraA back into the lipidic phase of the membrane during pilus retraction. Thus, the primary function of stoichiometrically bound lipids might be to lower that cost and thus lower the energetic barrier for assembly/disassembly in order to facilitate pilus dynamics. Testing such a hypothesis is clearly the next step in research in conjugative pilus biogenesis. Moreover, since there are other bacterial filaments that are assembled from subunits that exist at some point as integral membrane proteins (e.g., filamentous bacteriophage or type IV pili [not to be confused with T4S secretion pili]), it remains to be seen whether any of these are also lipoprotein filaments.

Bacteriophages have been used in the past and are still used widely in eastern Europe, notably Russia, to combat bacterial infections. In western Europe, their use declined rapidly when effective, cheap, and broad-range antibiotics became available. However, with antibiotics becoming increasingly ineffective, it has become urgent to explore all possible avenues in the search for novel therapeutic agents: phage therapy is poised to undergo a major revival as one potential weapon in the arsenal of antimicrobials. Effective treatment by bacteriophages will be greatly facilitated by a detailed characterization of the phage-pilus interaction at a molecular level. The structure presented here provides unprecedented atomic details of one interacting partner, the pilus, and we show that these can be exploited productively to switch the sensitivity to phage infection conferred by conjugative pili, providing a proof of concept that this knowledge can indeed be used to derive effective bacteriophage therapies to combat bacterial infectious diseases.

## STAR★Methods

### Key Resources Table

**Table:** 

REAGENT or RESOURCE	SOURCE	IDENTIFIER
**Chemicals, Peptides, and Recombinant Proteins**		

Common lab reagents	N/A	N/A

**Critical Commercial Assays**		

Quick & Easy *E.coli* gene deletion	Gene Bridges	Cat#K006

**Deposited Data**		

F-pilus pED208 Cryo-EM map	This study	EMD-4042
F-pilus pED208 structure	This study	5LEG
F-pilus (12.5Å axial rise) Cryo-EM map	This study	EMD-4046
F-pilus (12.5Å axial rise) structure	This study	5LFB
F-pilus (13.2Å axial rise) Cryo-EM map	This study	EMD-4044
F-pilus (13.2Å axial rise) structure	This study	5LER

**Experimental Models: Organisms/Strains**		

E.coli: JE2571 (*leu thr fla pil str*) harboring the pED208 plasmid	Lab of Prof. Ellen Zechner	N/A
*E. coli*: DH5α (F– Φ80*lac*ZΔM15 Δ(*lac*ZYA-*arg*F) U169 *rec*A1 *end*A1 *hsd*R17 (rK–, mK+) *pho*A *sup*E44 λ– *thi*-1 *gyr*A96 *rel*A1) harboring the pOX38-Cm plasmid	Lab of Prof. Fernando de la Cruz	N/A
f1 phage: ssDNA, filamentous	Lab of Dr. Neville Firth	N/A
HB101: auxotroph, F-, *recA13*, Res-, Mod-, Str^r^, Rif^r^	This study	N/A

**Recombinant DNA**		

Plasmid: pBAD-M11 (modified)	This study	N/A

**Sequence-Based Reagents**		

Primers for Kanamycin cassette generation, *traA* allele cloning and mutagenesis, see Table S1	This study	N/A

**Software and Algorithms**		

Spider package	Frank et al., 1996	http://spider.wadsworth.org/spider_doc/spider/docs/spider.html
CTFFIND3	Mindell and Grigorieff, 2003	http://grigoriefflab.janelia.org/ctf
EMAN2	Tang et al., 2007	http://blake.bcm.tmc.edu/emanwiki/EMAN2
IHRSR	Egelman, 2000	N/A
COOT	Emsley et al., 2010	http://www2.mrc-lmb.cam.ac.uk/personal/pemsley/coot/
PHENIX	Adams et al., 2010	https://www.phenix-online.org/documentation/index.html
CHIMERA	Pettersen et al., 2004	http://www.rbvi.ucsf.edu/chimera/
Molprobity	Chen et al., 2010	http://molprobity.biochem.duke.edu/
LSQKAB	Kabsch, 1976	http://www.ccp4.ac.uk/
PDBSET	Winn et al., 2011	http://www.ccp4.ac.uk/
PYMOL	Molecular Graphics System, Version 1.8 Schrödinger, LLC	https://www.pymol.org/

### Contact for Reagent and Resource Sharing

Contact should be directed to Gabriel Waksman at g.waksman@ucl.ac.uk or g.waksman@mail.cryst.bbk.ac.uk.

### Method Details

#### Pilus Production and Purification

The F pilus encoded by the pOX38 plasmid (a gift from Prof. Fernando de la Cruz) and the F-like pilus encoded by the pED208 plasmid (a gift from Prof. Ellen Zechner) were purified using the same protocol, from the surface of the DH5α and JE2571 cells, respectively. Cells were grown on six Luria-Bertani (LB) medium plates (25×25 cm) with no antibiotics for the production of pED208 pili but supplemented with 34 μg/mL Chloramphenicol (Cm) for production of F pili (pOX38) for 16 hr. Cells were scraped from each plate surface with 20 mL of SSC buffer (15 mM sodium citrate, 150 mM NaCl, pH 7.2) following incubation at 4°C for 2 hr with 1 L of the same buffer with gentle stirring. The suspension was centrifuged two times at 10,800 g for 20 min, and the supernatant was precipitated by adding 5% PEG6,000 and 500 mM of NaCl. After 2 hr of incubation at 4°C, the precipitate was collected by centrifugation of the suspension at 15,000 g for 30 min. The precipitate was resuspended in 120 mL of sterile water and centrifuged for 10 min at 5,000 g. The supernatant was precipitated again using the same conditions used previously, followed by centrifugation at 15,000 g for 20 min. At this stage, the purified pili were resuspended in 1 mL of 50 mM Tris-HCl, 200 mM NaCl, pH 8.0 (F pilus), and in PBS, pH 7.4 (pED208 pilus). Each suspension was layered on pre-formed CsCl step gradients (1.0–1.3 g/cm^3^) and centrifuged at 192,000 g for 17 hr at 4°C. The pili band was carefully removed and extensively dialysed against Tris-HCl, 200 mM NaCl, pH 8.0 (F pilus), or PBS, pH 7.4 (pED208 pilus). Pili purity was analyzed by SDS-PAGE, and the identification of TraA from both pili was verified by LC-ESI MS/MS.

#### Cryo-EM Sample Preparation and Data Collection

Each pilus sample (4μl) was applied to a glow-discharged Lacey 400 mesh copper grid (Agar Scientific). A Vitrobot plunge-freezing device (FEI) operating at 25°C and 100% humidity was used to incubate the sample with the grid for 1 min and blotting for 3.5 s prior to vitrification in liquid ethane. The data were collected on a FEI Tecnai G2 Polara operating at 300kV and equipped with a Gatan K2 Summit direct electron detector positioned at the end of a Quantum energy filter and an energy selecting slit width of 20 eV. The images were taken with a total dose of ∼100 e^−^/Å^2^ fractionated over 60 frames with a calibrated pixel size of 1.1Å/pixel. Images were taken within a defocus range of −0.5 to −3.5 μm.

#### Cryo-EM Image Processing and Reconstruction

The Spider software package (Frank et al., 1996) was used for most operations, unless otherwise noted. The program CTFFIND3 (Mindell and Grigorieff, 2003) was used for determining defocus values. For the pED208 filaments, 362 images were used after removing those with drift, a poor contrast transfer function (CTF), or a defocus greater than 3.0 μm. The images were corrected for phase reversals by multiplying them by the calculated CTF, which is a Wiener filter in the limit of a very poor SNR. The program e2helixboxer from the EMAN2 suite (Tang et al., 2007) was used for extracting images of long filaments, and 3,841 long boxes were selected. From these, 43,952 overlapping boxes (each 384 px long, and each shifted 18 px, or ∼1.5 times the axial rise per subunit) were cut. The boxing of filaments and CTF estimation was done using the total dose, while the subsequent processing was done with the boxes cut from the frames with a dose of 20 electrons/Å^2^. The Iterative Helical Real Space Reconstruction (IHRSR) method (Egelman, 2000) was used for the reconstruction. Several possible helical symmetries were investigated, but only the one with a C5 rotational point group symmetry yielded recognizable α helices. Once the structure was stable and further iterations introduced no changes, the parameters (Euler angles, x- and y-shifts) found for the 20 electrons/Å^2^ boxes were applied to the 10 electrons/Å^2^ boxes to reduce radiation damage in the final reconstruction. The CTF was corrected by dividing the volume by the sum of the squared CTFs, since the images had been multiplied by the CTF twice: once by the microscope, and once when phases were corrected.

The same approach was used for the F pilus filaments, with 297 images being selected for further processing. From these, 1,259 long boxes were extracted, from which 28,395 overlapping boxes (each 384 px long) were cut. In contrast to the pED208 filaments, power spectra from the F pili showed two symmetries present, which discretely differed in terms of the axial rise per subunit. Several cycles of sorting were used to separate the segments into two classes. Initially, a reconstruction from the unsorted set was used to generate two reference volumes, one with an axial rise of ∼12.5 Å and the other with ∼13.2 Å. Two separate reconstructions were generated from each of these two sets, and these were then used as new references to once again sort the entire dataset. The dataset used for the 12.5 Å reconstruction contained 16,426 segments, while the 13.2 Å set contained 11,969 segments. Due to the limited resolution in the F pili reconstructions, the 20 electrons/Å^2^ images were used for the final reconstruction, as nothing would be gained using a lower dose.

#### Model Building and Refinement

The initial model for a single pilin chain was built using COOT (Emsley et al., 2010) followed by iterative rounds of real space refinement and building using PHENIX (Adams et al., 2010) and COOT, respectively. During initial model building of the pED208 pilin, an extra density was noticed which was clearly not part of the pilin TraA. Investigation of this extra density suggested it could be a single phospholipid. MS analysis indicated the presence of two PG species, PG 32:1(16:0, 16:1) and PG 34:1 (16:0, 18:1), and thus PG 32:1 was modeled in the density using COOT and refined with PHENIX. In the case of the F pilus, only the PG head group was modeled as density for the aliphatic chains was not visible. In all cases, the initial coordinates required for modeling the phospholipid were generated using COOT’s “Ligand builder” tool, and the dictionary file required for refinement were generated using the CCP4 “Make ligand” tool (Debreczeni and Emsley, 2012). Progress in refinement was tracked through Ramachandran plot and Molprobity (Chen et al., 2010). Once a single unit (pilin TraA and phospholipid PG) was successfully refined, a pdb coordinate file with two TraA chains was generated by fitting two individual TraA chains within a pilus strand using CHIMERA (Pettersen et al., 2004). The rotation and translation parameters by which these two chains were related were then calculated using the program LSQKAB (Kabsch, 1976). These parameters were used to generate a strand of the pilus, each containing 16 single-TraA-PG units using the CCP4 program PDBSET (Winn et al., 2011). In order to build a pilus from a single strand set of coordinates, a pdb coordinate file with two TraA chains was generated by fitting two individual TraA chains in adjacent strands using CHIMERA. This file was used to calculate the rotation and translation parameters again using the program LSQKAB. These values representing the relation between two adjacent strands were used to build the entire five-stranded pilus using the program PDBSET.

#### Mass Spectrometry Analysis of Lipids

Lipid extractions from purified pili were achieved by three successive vigorous extractions with ethanol (90% v/v) (Fyffe et al., 2006). The pooled extracts were dried by nitrogen gas in a glass vial and re-extracted using a modified Bligh and Dyer method (Richmond et al., 2010). For whole-cell control, membranes were washed with PBS and lipids were extracted following the same procedure. Pili were treated with Phospholipase A2 (0.1 units) in PBS for 16 hr at 37°C followed by heat inactivation and extraction as described above.

Extracts were dissolved in 15 μL of chloroform:methanol (1:2) and 15 μL of acetonitrile:propan-2-ol:water (6:7:2) and analyzed with a Absceix 4000 QTrap, a triple quadrupole mass spectrometer equipped with a nano-electrospray source. Samples were delivered using a Nanomate interface in direct infusion mode (∼125 nL/min). Lipid extracts were analyzed in both positive and negative ion modes using a capillary voltage of 1.25 kV. MS/MS scanning (daughter, precursor, and neutral loss scans) was performed using nitrogen as the collision gas with collision energies between 35–90 V.

#### Construction of Mutants

The *tra*A gene in pED208 was disrupted from the native pED208 (in the JE2571 strain) using the Quick & Easy *E. coli* gene deletion kit (Gene Bridges) protocol. This protocol resulted in a mutant (termed “pED208_ΔTraA”) where the *tra*A gene was disrupted by a Kanamycin (Km)-resistance cassette. Positive recombinants were selected on LB plates supplemented with 15 μg/mL Km, and the correct location of the recombination event was confirmed by sequencing with suitable primers. The WT and mutated *traA* genes were cloned into a modified pBADM-11 vector (which confers Carbenicillin [Cb] resistance and is inducible using L-arabinose) where the His-tag was removed using conventional molecular cloning and site directed mutagenesis protocols. All primers used for *tra*A gene disruption, cloning, and mutants generation are described in Table S1.

#### Negative Stain Electron Microscopy

To assay for pilus expression, pED208_ΔTraA complemented with the WT *tra*A or mutants were grown in LB medium supplemented with 15 μg/mL Km and 50 μg/mL Cb to an OD_600_ of 0.5 and were induced using 0.05% L-arabinose until an OD_600_ of 1.5. 10 μL of bacterial cultures were then deposited for 2 min on a glow-discharged 400 mesh carbon-coated cooper grid (Agar Scientific). The grid was then washed with two drops of water and stained for 10 s with 0.2% w/v of phosphotungstic acid (PTA). Images were taken with a Gatan CCD camera on a Tecnai electron microscope (FEI) operating at 120 kV.

#### Phage Sensitivity Assay

Sensitivity (S) or resistance (R) of bacteria to filamentous phage f1 (a gift from Dr. Neville Firth) was determined qualitatively using a spot phage test. Cells containing pED208_ΔTraA complemented with either the WT *tra*A or each of the pilin mutant constructs were induced at an OD_600_ of 0.5 using 0.05% L-arabinose and grown to a final OD_600_ of 1.5 before being plated onto LB plates containing 50 μg/ml Cb, 15 μg/mL Km, and 0.05% L-arabinose and onto which a 20 μL aliquot of phage (1x10^8^ pfu) was spotted. After the agar surface had dried, the plates were incubated overnight to allow plaque lytic development.

#### Conjugation Assay

Mid-exponential phase cultures (OD_600_ of one, which was equivalent to 5.5 × 10^8^ cells per mL) were used for conjugation experiments by the quantitative filter-mating method. In brief, aliquots (0.5 mL) of donor (JE2571 containing pED208_ΔTraA complemented with WT and mutant TraA induced as above) and recipient (Rifampicin (Rif)-resistant HB101) cultures were mixed and filtered through a nitrocellulose membrane filter (Sartorius; 0.45 μm pore size), which was then placed onto the surface of a LB plate and incubated at 37°C for 2 hr. After incubation, the bacteria on the filter were suspended in 2 mL of LB, serially diluted (10-fold) in saline and 0.1 mL aliquots of the dilutions plated onto LB medium with 100 μg/mL Rif and 15 μg/mL Km and incubated overnight. Conjugative transfer efficiencies reported as ratios of trans-conjugants per donor were then derived. Experiments were performed three times.

### Quantification and Statistical Analysis

Quantification and statistical analyses employed in this publication pertain to the analysis on electron microscopy data and the determination of structures by electron microscopy, which are integral parts of existing algorithms and software used.

### Data and Software Availability

#### Data Resources

All data were deposited in EMDB and PDB with the following entry codes: EMDB: EMD-4042 and PDB: 5LEG (F-pilus pED208); EMDB: EMD-4046 and PDB: 5LFB (F-pilus 12.5 Å axial rise); EMDB: EMD-4044 and PDB: 5LER (F-pilus 13.2 Å axial rise).

## Author Contributions

T.R.D.C. purified pili, prepared cryo-EM grids, collected NS-EM pictures, extracted lipids from membranes and pili, and carried out the mutagenesis work and biological assays together with J.M.S. A.I. built and refined all models. M.U. and A.R. collected cryo-EM data. J.M.S. supervised phage and conjugation experiments. T.K.S. carried out MS experiments and analyzed the results. E.H.E. performed the image analysis and carried out the EM reconstructions. G.W. supervised the work, made figures, and wrote the manuscript.

## Supplemental Information

Table S1. Oligonucleotides Used in this Study, Related to STAR Methods “Construction of Mutants” Section Table S1. Oligonucleotides Used in this Study, Related to STAR Methods “Construction of Mutants” Section
