# Supplementary material for: Structure of the Bacterial Sex F Pilus Reveals an Assembly of a Stoichiometric Protein-Phospholipid Complex
Source: Cell. 2016 Sep 8;166(6):1436–1444.e10. doi: 10.1016/j.cell.2016.08.025 (PMC5018250; doi:10.1016/j.cell.2016.08.025)
Supplement: Table S1. Oligonucleotides Used in this Study, Related to STAR Methods “Construction of Mutants” Section [file mmc1.pdf]

Cell, Volume *166*

Supplemental Information

Structure of the Bacterial Sex F Pilus Reveals an Assembly of a Stoichiometric Protein-Phospholipid Complex

Tiago R.D. Costa, Aravindan Ilangovan, Marta Ukleja, Adam Redzej, Joanne M. Santini, Terry K. Smith, Edward H. Egelman, and Gabriel Waksman

Table S1 – Oligonucleotides used in this study (related to METHODS "Construction of mutants" section).

Purpose	Oligonucleotide name and sequence
Kanamycin cassette generation	TraA_KO2_F 5' – ATGAATTTATCCTTTGCAAAAGGCGGCCTCCCTG CGCCTGTAAAAAACCGAATTAACCCTCACTAAAGGGCGG<br>TraA_KO2_R 5' – TCATTTGATGAAGGTAAGACCGACGGTAGTGAAGA CGATAACCACAACCATAATACGACTCACTATAGGGCTCG
traA allele cloning into modified pBAD-M11	TraAinBAD_F 5' – CAGGAGGAATTAACCATGAATTTATCCTTTGCA AAAGG<br>TraAinBAD_R 5' – AGCCAAGCTCTCTTATCATTTGATGAAGGTAA GACCG
traA mutagenesis	Y37F_F 5' – GCGATGTTCATCCGCACCAAGAACC
	Y37F_R 5' – GCGGATGAACATCGCCACACCGAC
	Y37V_F 5' – GCGATGGTTATCCGCACCAAGAACC
	Y37V_R 5' – GCGGATAACCATCGCCACACCGAC
	A28F_F 5' – ATCATTTTCGAACTGATTGTCGGTGTGG
	A28F_R 5' – CAGTTCGAAAATGATGATACACATCATGACG
	A28N_F 5' – ATCATTAACGAACTGATTGTCGGTGTGG
	A28N_R 5' – CAGTTCGTTAATGATGATACACATCATGACG
	R39E_F 5' – TATATCGAAACCAAGAACCTGCTGATCC
	R39E_R 5' – CTTGGTTTCGATATACATCGCCACACCGAC
	R39A_F 5' – TATATCGCCACCAAGAACCTGCTGATCC
	R39A_R 5' – CTTGGTGGCGATATACATCGCCACACCGAC
